# High prevalence of knockdown resistance mutations, genetic clade diversity, and detection of *Acinetobacter* species in head lice (*Pediculus humanus capitis*) infesting children in a Thai orphanage: A comprehensive survey

**DOI:** 10.1016/j.crpvbd.2025.100336

**Published:** 2025-11-17

**Authors:** Urooj Gul, Sakone Sunantaraporn, Narisa Brownell, Padet Siriyasatien

**Affiliations:** a Medical Science Program, Faculty of Medicine, Chulalongkorn University, Bangkok, 10330, Thailand; b Center of Excellence in Vector Biology and Vector-Borne Diseases, Department of Parasitology, Faculty of Medicine, Chulalongkorn University, Bangkok, 10330, Thailand; c Department of Parasitology, Faculty of Medicine, Chulalongkorn University, Bangkok, 10330, Thailand

**Keywords:** *Pediculus humanus capitis*, *Acinetobacter*, Genetic diversity, Knockdown resistance, Orphanage, Thailand

## Abstract

Pediculosis, caused by the head louse (*Pediculus humanus capitis*), remains a public health challenge among school-aged children. The emergence of resistance to pyrethroid insecticides associated with mutations in the knockdown resistance (*kdr*) genes may hinder control efforts. Furthermore, head lice have been implicated as potential vectors for bacterial pathogens, raising concerns about their role in disease transmission. Head lice collected from children in an orphanage were analyzed for genetic diversity, *kdr*-mutation resistance, and the presence of bacterial pathogens. A total of 107 lice samples were screened for the *kdr* T917I mutation using PCR-RFLP. Clade diversity was assessed through phylogenetic analysis of *cytb* gene sequences, supported by species delimitation methods. Detection of bacterial pathogens, including *Acinetobacter* spp. and *Bartonella* spp., was conducted using PCR targeting the *rpoB* and *gltA* genes, respectively. Phylogenetic reconstruction and species delimitation analyses consistently revealed the presence of two distinct genetic clades among the head lice samples: Clade A (69.16%) and Clade C (30.84%). The homozygous resistant (RR) genotype was predominant, observed in 101 individuals (94.39%), with an overall *kdr* T917I resistance allele frequency of 96%. Molecular screening for bacterial pathogens identified *Acinetobacter towneri*, *A. johnsonii*, and *Acinetobacter* spp. in three samples, while no *Bartonella* spp. were detected. The study identified significant pyrethroid resistance among head lice in the studied population and a notable presence of *Acinetobacter* species. This underscores the necessity for ongoing surveillance, especially among vulnerable populations such as children in orphanages.

## Highlights

• Two genetic clades (Clade A and Clade C) of head lice were identified in orphanage children.
• High prevalence of *kdr* mutation was detected: 94.4% homozygous resistant (RR) with a T917I allele frequency of 96%.
• *Acinetobacter towneri*, *A. johnsonii*, and *Acinetobacter* sp. were detected in 2.8% of the lice samples.
• Molecular surveillance of head lice in orphanage children underscores the necessity for alternative control strategies.

## Graphical abstract

**Figure:**
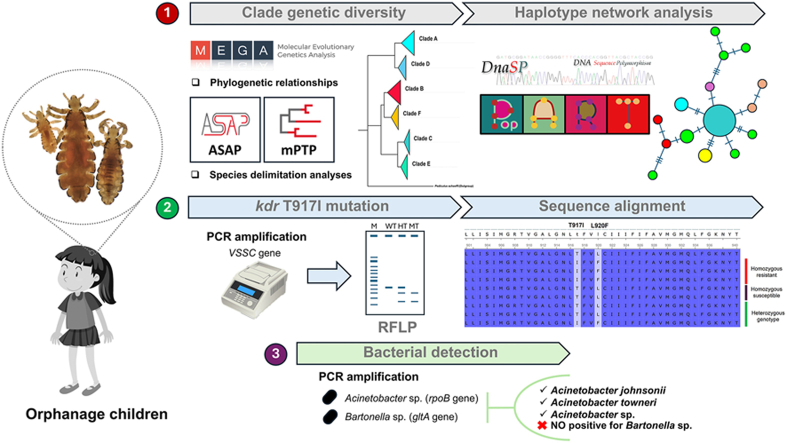

## 1 Introduction

*Pediculus humanus capitis*, commonly known as the human head louse, is an obligate ectoparasite that feeds exclusively on human blood and resides on the scalp (Fu et al., 2022). Head lice infestation, or pediculosis capitis, remains a pervasive public health concern globally, particularly among school-aged children and populations living under crowded or unhygienic conditions (Falagas et al., 2008). Orphanage children, especially those in institutional care settings, represent a vulnerable group with increased risk due to close-contact living environments (Mahmud et al., 2011; Cummings et al., 2018).

Head lice are often viewed as a nuisance, but recent molecular studies have shown their potential role as carriers of harmful bacteria. These findings suggest that head lice might help spread bacterial pathogens, especially in crowded and unhygienic settings. In controlled laboratory settings, head lice have been shown to have the capacity to transmit *Rickettsia prowazekii* (Robinson et al., 2003). Nevertheless, they have not been definitively identified as vectors in outbreaks of louse-borne epidemic typhus, which is traditionally associated with the body louse. Head lice have been found to contain DNA from several medically important bacteria, including *Acinetobacter* spp., *Staphylococcus aureus*, *Serratia marcescens*, *Bartonella quintana*, *Coxiella burnetii*, and *Rickettsia aeschlimannii* (Sunantaraporn et al., 2015; Amanzougaghene et al., 2017; Mokhtar et al., 2020; Dzul-Rosado et al., 2022). Growing molecular evidence challenges the long-held belief that head lice are non-threatening parasites and highlights the necessity to reevaluate their potential role in bacterial transmission dynamics. It is important to know if head lice are only passive carriers of bacterial DNA or active reservoirs with vectorial potential for understanding their true significance to human health (Amanzougaghene et al., 2019b).

Advances in molecular analyses have significantly deepened our understanding of the genetic diversity within head lice clades (Light et al., 2008; Amanzougaghene et al., 2016, 2017, 2019a, 2020; Louni et al., 2018). In particular, mitochondrial gene sequencing has been advantageous in exposing the substantial heterogeneity that exists among global populations. Mitochondrial DNA analysis has revealed six genetically distinct clades (A, B, C, D, E, and F) of body lice (*P. h. humanus*) and head lice (*P. h. capitis*), each exhibiting unique geographical distribution and possible associations with specific human host populations (Light et al., 2008; Sunantaraporn et al., 2015; Amanzougaghene et al., 2019a). Haplotype A5 from clade A and haplotype B36 from clade B are the most prevalent haplotypes worldwide, evidence of their epidemiological significance and pervasive distribution (Amanzougaghene et al., 2016, 2020). In Thailand, genetic diversity among head lice collected from primary school children has been documented, with clades A and C identified based on sequences of the cytochrome *c* oxidase subunit 1 (*cox*1) and cytochrome *b* (*cytb*) genes (Sunantaraporn et al., 2015; Phadungsaksawasdi et al., 2021; Yingklang et al., 2021). However, genetic data remain limited, particularly for infestations affecting underrepresented and vulnerable populations such as orphanage children.

Control of head lice traditionally relies on pyrethroid-based insecticides, notably permethrin. However, treatment failures are increasingly reported due to the emergence of knockdown resistance (*kdr*) mutations in the voltage-sensitive sodium channel (*VSSC*) gene of lice (Yingklang et al., 2023; Brownell et al., 2024). Pyrethroid resistance in Thai head lice, associated with the *kdr* mutation, increased from negligible prevalence in 2010 to over 60% by 2021. Key mutations, including M815I, T917I, and L920F, reduce the sensitivity of the sodium channel to pyrethroids, undermining treatment efficacy and complicating control efforts (Lee et al., 2000, 2003; Hodgdon et al., 2010; Brownell et al., 2020).

In this study, we aim to identify mitochondrial clades based on *cytb* sequences, detect *kdr* T917I mutations associated with pyrethroid resistance, and screen for bacterial pathogens, including *Acinetobacter* spp. and *Bartonella* spp., in head lice collected from orphanage children in Ang Thong Province, Thailand. These findings provide preliminary insights into the epidemiological patterns of head lice among vulnerable communities and may guide future evidence-based strategies for control and public health management.

## 2 Materials and methods

### 2.1 Participant recruitment and head louse sample collection

Children residing in an orphanage in Ang Thong Province, central Thailand, aged 7–12 years at the time of enrollment, were included in the study if head lice infestation was confirmed through scalp examination. Exclusion criteria included being outside the designated age range, having used lice treatment products (such as lotions, creams, sprays, or prescription medications) within the two weeks before enrollment, the presence of scalp conditions like severe dandruff or open wounds, or lack of consent to participate. Head lice samples, including adult females, males, nymphs, and eggs, were collected from schoolchildren. The lice were carefully removed from the hair using fine-toothed combs, placed into sterile containers, and preserved in 70% ethanol. All collected samples were then transported to the Center of Excellence in Vector Biology and Vector-Borne Diseases, Department of Parasitology, Faculty of Medicine, Chulalongkorn University, for further investigation.

### 2.2 DNA extraction

To prevent external contamination, the surface of each louse was decontaminated prior to DNA extraction, following previously established protocols (La Scola et al., 2001). A total of 107 individual head lice were washed three times with sterilized distilled water. Each louse was then homogenized using a sterile plastic pestle in 200 μl of cell lysis buffer, followed by the addition of 20 μl of proteinase K solution. The samples were incubated at 56 °C for 16 h. Genomic DNA was extracted using the DNA Extraction Kit (GeneAll Biotechnology Co., Ltd., Seoul, Korea), according to the manufacturer’s instructions. The head lice genomic DNA was eluted in 50 μl of pre-warmed elution buffer. The concentration and purity of the extracted DNA were assessed using a Nanodrop spectrophotometer (Thermo Scientific, USA). The samples were then stored at −20 °C until further analysis.

### 2.3 Genotyping of the head lice clade based on *cytb* gene amplification

The mitochondrial cytochrome *b* (*cytb*) gene was selected as a genetic marker for clade identification of head lice using conventional PCR. A 348-bp fragment of the *cytb* gene was amplified using primers described in a previous study (Li et al., 2010). PCR conditions followed the protocol established by Phadungsaksawasdi et al. (2021). The resulting PCR products were purified using the FavorgenPrep™ GEL/PCR Purification Kit (Favorgen, Ping Tung, Taiwan). Sanger sequencing was conducted by Macrogen Inc. (South Korea) using the same forward and reverse primers employed for *cytb* amplification. The *cytb* sequences were subsequently compared to existing sequences in GenBank using the Basic Local Alignment Search Tool (BLAST) available at https://blast.ncbi.nlm.nih.gov/Blast.cgi. The newly generated sequences were deposited in the GenBank database under the accession numbers PX505907-PX505921.

To identify head lice clades, an integrative taxonomic approach was employed, incorporating phylogenetic analysis and species delimitation methods. Phylogenetic reconstruction was conducted using the maximum likelihood (ML) method with 1000 bootstrap replicates, implemented in MEGA 11 (Tamura et al., 2021). Species delimitation was carried out using two approaches, Assemble Species by Automatic Partitioning (ASAP) (Puillandre et al., 2021) and the multi-rate Poisson Tree Processes (mPTP) (Kapli et al., 2017). The ASAP analysis was performed using three substitution models, p-distance, Kimura 2-parameter (K80), and Jukes-Cantor (JC69). In contrast, mPTP analysis was conducted *via* the mPTP web server (https://mptp.h-its.org/#/tree) using the default settings. This method estimates species boundaries based on maximum likelihood derived from the phylogenetic tree and provides support values for each delimited clade. Intraspecific genetic divergence was assessed using the Kimura 2-parameter (K2P) model implemented in MEGA 11 (Tamura et al., 2021).

### 2.4 Haplotype network and genetic diversity of the head lice population

Haplotype network analysis of *cytb* sequences was performed using data from previously published studies on head lice populations in Thailand (Phadungsaksawasdi et al., 2021; Yingklang et al., 2021), along with sequences generated in the present study. Sequence data were analyzed in DnaSP version 6 (Rozas et al., 2017), and haplotype networks were constructed using the median-joining method in PopART version 1.7 (Bandelt et al., 1999; Leigh and Bryant, 2015). Genetic diversity indices including the number of haplotypes (*H*), number of polymorphic sites (*S*), average number of nucleotide differences (*κ*), haplotype diversity (*Hd*), and nucleotide diversity (*π*) were calculated using DnaSP v.6. Neutrality tests were also conducted based on Tajima’s *D* and Fu and Li’s *D* statistics to assess potential deviations from neutral evolution (Fu, 1997; Tajima, 1989; Rozas et al., 2017).

### 2.5 Identification of knockdown (*kdr*) resistance mutation based on PCR-RFLP

Conventional PCR was used to amplify a 332-bp fragment of the α-subunit of the voltage-sensitive sodium channel (*VSSC*) gene, which includes the *kdr* T917I mutation, using specific primers described by Durand et al. (2007). The PCR thermal cycling conditions followed the protocol outlined by Brownell et al. (2020). To screen for the *kdr* mutation, PCR products were subjected to restriction fragment length polymorphism (RFLP) analysis using the *Ssp*I restriction enzyme (New England Biolabs, Ipswich, MA, USA), which detects the T917I mutation (A**C**A > A**T**A). Digested fragments were separated on a 2% agarose gel *via* electrophoresis at 90 V for 50 min. Gels were then stained with ethidium bromide and visualized using a Gel Doc EQ system (Bio-Rad, Hercules, CA, USA). The presence of the T917I mutation was determined based on distinct RFLP banding patterns: the homozygous susceptible genotype (SS) demonstrated a single 332-bp band, the homozygous resistant genotype (RR) showed two bands (at 261 bp and 71 bp), and the heterozygous genotype (RS) presented three bands (at 332 bp, 261 bp, and 71 bp), as described by Brownell et al. (2020). Sanger sequencing was conducted on the PCR products to confirm the presence of homozygous susceptible (SS), heterozygous (RS), and homozygous resistant (RR) genotypes. Sequencing was performed using the same forward and reverse primers targeting the *VSSC* gene by a commercial service provider (Macrogen, Seoul, South Korea). The *VSSC* sequences generated in this study were deposited in the GenBank database under the accession numbers PX505922-PX505929. Nucleotide and corresponding amino acid sequences were analyzed and compared using Unipro UGENE software version 51.0 (Okonechnikov et al., 2012).

The frequencies of *kdr* T917I genotypes (RR, RS, and SS) were calculated by dividing the number of individuals with each genotype by the total number of head lice analyzed. These genotype frequencies were then evaluated for deviation from Hardy-Weinberg equilibrium using a chi-square (*χ*^2^) test, and Wright’s inbreeding coefficient (*F*_IS_) was also calculated to assess potential inbreeding within the population (Black and Krafsur, 1985; Brownell et al., 2020).

### 2.6 Detection of bacterial pathogens in head lice DNA

To identify bacterial pathogens, conventional PCR was employed to amplify the *gltA* gene for *Bartonella* spp. (Norman et al., 1995) and the *rpoB* gene for *Acinetobacter* spp. (Kempf et al., 2012), following the protocols described by Sunantaraporn et al. (2015) and Promrangsee et al. (2019). The positive PCR products were ligated into the pGEM-T Easy Vector (Promega, Madison, WI, USA) and transformed into *Escherichia coli* DH5α competent cells. The transformed cells were incubated at 37 °C for 16–18 h on Luria-Bertani (LB) agar plates supplemented with ampicillin, IPTG, and X-Gal to facilitate blue-white screening. White colonies were selected and verified for the presence of inserts *via* colony PCR. Five confirmed colonies were then cultured in LB broth containing ampicillin. Plasmid DNA was extracted using the GeneAll® Exprep™ Plasmid Purification Kit (GeneAll Biotechnology Co., Ltd., Seoul, Korea) according to the manufacturer’s protocol. Sanger sequencing was performed using T7 promoter primers by Macrogen Inc. (Seoul, Korea). The newly generated *rpoB* sequences were submitted to the GenBank database under the accession numbers PX505930 (*Acinetobacter johnsonii*), PX505931 (*Acinetobacter* sp.), and PX505932 (*Acinetobacter towneri*).

## 3 Results

### 3.1 Genetic clade diversity of head lice

**Fig. 1:**
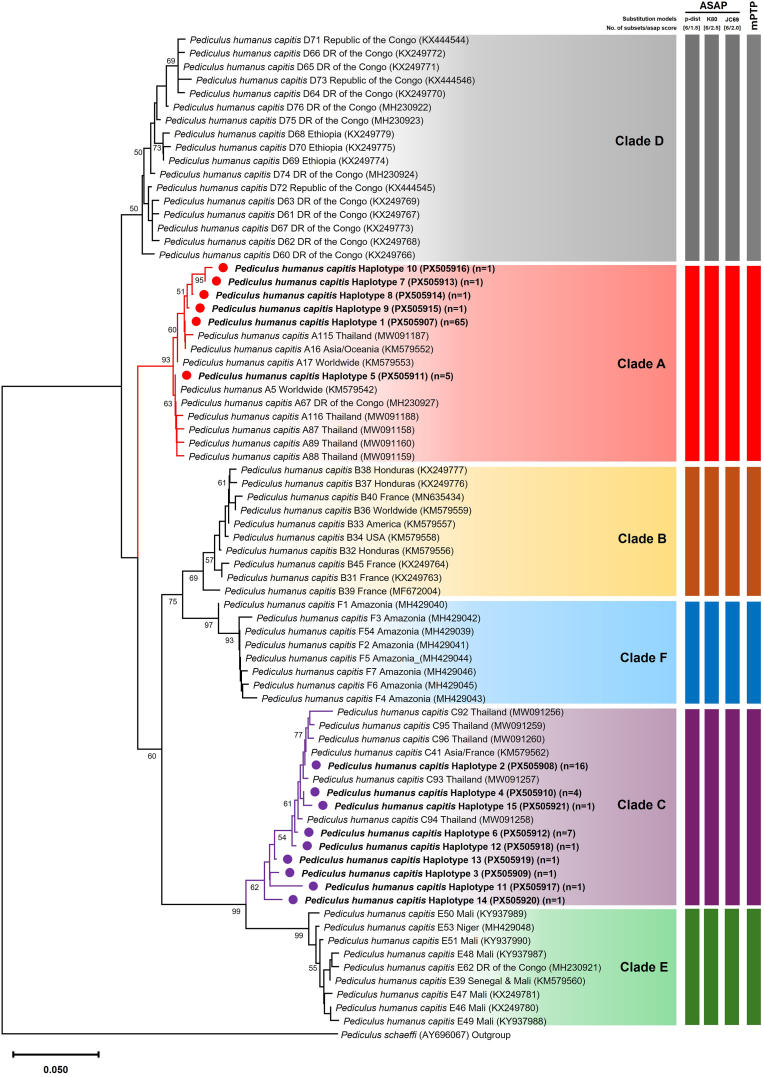
Maximum likelihood phylogenetic tree depicting the relationships among head lice clades identified in this study, constructed using the Hasegawa-Kishino-Yano model with a gamma distribution (HKY+G) and 1000 bootstrap replicates. The scale-bar represents 5% nucleotide sequence divergence. Vertical bars adjacent to the tree indicate species delimitation results based on the ASAP and mPTP methods. Red and purple circles indicate sequences generated in the present study.

**Fig. 2:**
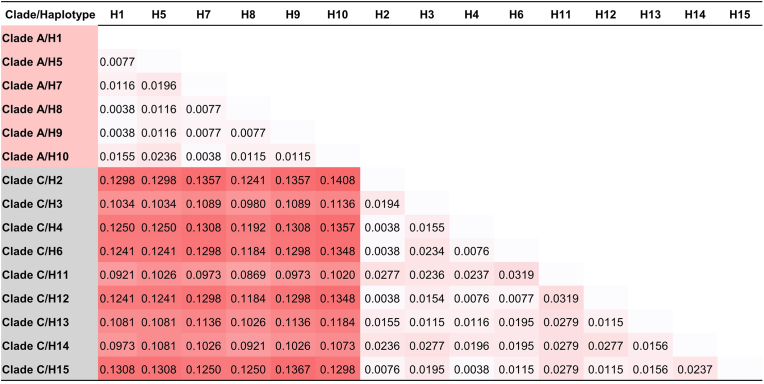
Intraspecific genetic divergence based on *cytb* gene sequences from Clade A and Clade C identified in head lice in the present study.

A total of 464 head lice, including 90 females, 135 males, and 239 nymphs, were collected from 107 orphanage children, with an average infestation rate of 8.5 lice per individual. Head lice infestation was detected in 25.5% (107/420) of participants, with a prevalence of 50.5% (107/212) in girls and no occurrences in boys (0/208). DNA was extracted from each of the 107 (female, *n* = 45; male, *n* = 46; and nymph, *n* = 16) samples and analyzed using PCR targeting the *cytb* gene to determine clade identification. BLAST analysis showed that all sequences matched *Pediculus humanus capitis*, with sequence similarity ranging from 97.36 to 100%. An integrative taxonomic approach, incorporating phylogenetic reconstruction and species delimitation methods (ASAP and mPTP), was applied to confirm clade differentiation. Both the phylogenetic analysis and species delimitation algorithms consistently identified two genetically distinct clades among the head lice samples: Clade A (*n* = 74; 69.16%) and Clade C (*n* = 33; 30.84%) (Fig. 1). The intraspecific genetic divergence within each clade, based on the Kimura 2-parameter (K2P) model, was relatively low, ranging from 0.38 to 2.36% for Clade A and from 0.38 to 3.19% for Clade C. In contrast, the interclade divergence between clades A and C was substantially higher, exceeding 3%, with pairwise distances ranging from 9.21 to 14.08% (Fig. 2).

### 3.2 Haplotype network analysis and genetic diversity in the head lice population

**Fig. 3:**
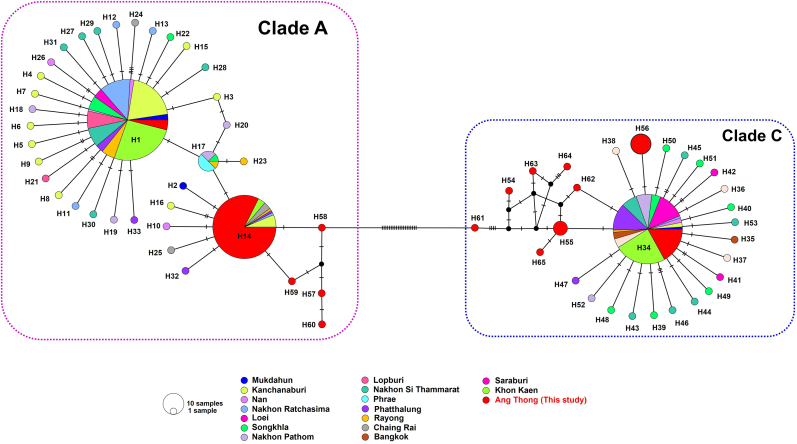
*cytb* haplotype networks of human head lice from Thailand, including samples collected in the present study. Each circle represents a unique haplotype, with the size of the circle proportional to its frequency. The colored segments within the circles correspond to different provinces, indicating the geographical origin and the number of sequences per haplotype. Hatch marks on the lines connecting the circles represent mutational steps between haplotypes.

This study explores the genetic diversity of head lice using a dataset of 381 *cytb* gene sequences obtained from both newly collected samples and previously published data across 16 provinces in Thailand. A total of 65 haplotypes were identified. Among these, the sequences predominantly clustered into three major haplotypes, H1, H14, and H34, alongside reference sequences from different areas in Thailand. A star-like or complex distribution pattern was observed, involving the origin haplotype and several connecting haplotype lineages that share a few base mutations (Fig. 3). Additionally, four and seven of our sequences shared identical haplotypes, corresponding to H55 and H56, respectively. The remaining 10 haplotypes were unique. According to the results of genetic diversity and neutrality statistics of head lice samples identified in Thailand, the haplotype diversity (*Hd*) was relatively high (0.781 ± 0.012), while nucleotide diversity (*π*) was comparatively low (0.0504 ± 0.0015). Neutrality tests indicated a non-significant Tajima’s *D* value (0.2160) and a significantly negative Fu and Li’s *D* value (−6.5423). Compared with the results of the present study, the haplotype diversity (*Hd*) was relatively high (0.606 ± 0.051), whereas the nucleotide diversity (*π*) remained low (0.0454 ± 0.0036). Neutrality tests yielded a significantly positive Tajima’s *D* (2.9612) and Fu and Li’s *D* (1.662), indicating a potential signature of balancing selection within the population (Table 1).

**Table 1:** Genetic diversity and neutrality statistics of *cytb* gene haplotypes of head lice identified in Thailand, and the present study.

Sample	*n*	Genetic diversity					Neutrality tests	
		No. of haplotypes (*H*)	No. of polymorphic sites (*S*)	Average no. of nucleotide differences (*κ*)	Haplotype diversity (*Hd* ± SD)	Nucleotide diversity (*π* ± SD)	Tajima’s *D*	Fu and Li’s *D*
Thailand	381	65	79	13.3446	0.781 ± 0.012	0.0504 ± 0.0015	0.2160 (*P* = 0.829)	−6.5423 (*P* < 0.0001)∗∗
Present study	107	15	32	12.0451	0.606 ± 0.051	0.0454 ± 0.0036	2.9612 (*P* = 0.0008)∗∗	1.6624 (*P* = 0.016)∗

*Note*: ∗*P* < 0.05, ∗∗*P* < 0.001.

*Abbreviations*: *n*, number of sequences; SD, standard deviation.

**Fig. 4:**
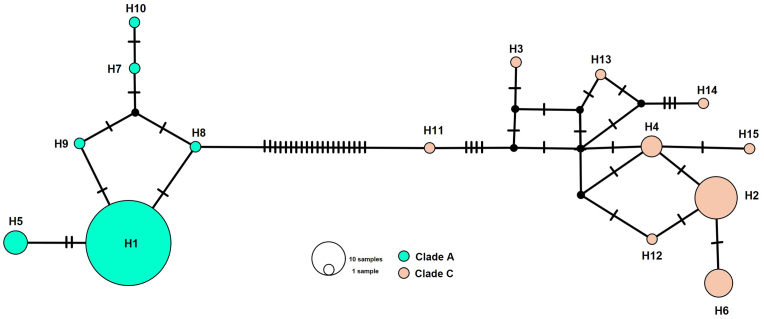
Haplotype network based on 107 *cytb* sequences of head lice collected in this study. The size of each circle corresponds to the number of individuals sharing that haplotype. Hatch marks on the lines connecting the circles represent the number of nucleotide substitutions separating the haplotypes.

**Fig. 5:**
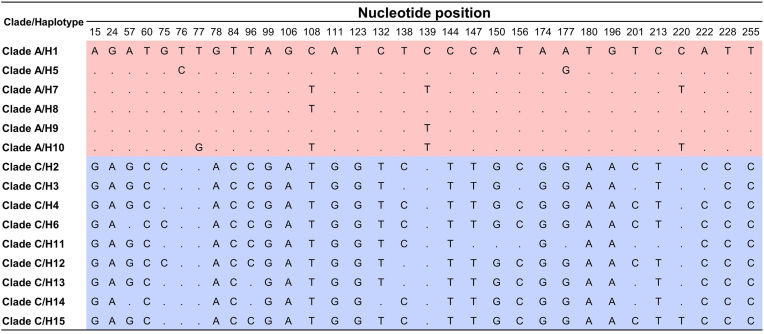
Sequence alignment illustrating the 32 polymorphic sites in the *cytb* gene between sequences of head lice Clade A and Clade C (265 bp).

Genetic diversity and haplotype network analyses were also conducted based on the newly generated 107 *cytb* sequences. A total of 15 haplotypes were identified and distributed across two mitochondrial Clades: Clade A, comprising six haplotypes (H1, H5, H7, H8, H9, and H10), and Clade C, comprising nine haplotypes (H2, H3, H4, H6, H11, H12, H13, H14, and H15) (Fig. 4). A total of 32 polymorphic nucleotide positions were observed in the *cytb* sequences between head lice Clades A and Clade C, with transition mutations accounting for the majority of these sites (e.g. A↔G or C↔T) (Fig. 5).

### 3.3 *kdr* (T917I) resistance mutation

In this study, restriction fragment length polymorphism (RFLP) analysis was employed to screen for the *kdr* T917I mutation. This method distinguished among three genotypes based on distinct RFLP patterns: homozygous susceptible or wild type (SS), heterozygous (RS), and homozygous resistant or mutant (RR). In total, 107 head lice samples were genotyped for the *kdr* mutation. The homozygous resistant (RR) genotype was the most prevalent, detected in 101 individuals (94.39%), followed by the heterozygous (RS) genotype in 4 individuals (3.74%), and the homozygous susceptible (SS) genotype in 2 individuals (1.87%). Within Clade A (*n* = 74), 70 lice (94.59%) exhibited the RR genotype, and 4 (5.41%) were identified as RS, with no SS genotype detected in this clade. In contrast, among the 33 samples from Clade C, 31 individuals (93.94%) carried the RR genotype, 2 (6.06%) carried the SS genotype, and the RS genotype was absent. The gel electrophoresis images obtained from the RFLP analysis are provided in Supplementary Fig. S1.

Overall, the frequency of the *kdr* T917I resistance allele was 0.96 across all 107 head lice samples. When analyzed by clade, the resistance allele frequency was 0.97 in Clade A and 0.94 in Clade C. Analysis using the Hardy-Weinberg model indicated that the overall distribution of *kdr* genotype frequencies in the population did not significantly deviate from expected values (Table 2). However, when analyzed by clade, only the head lice in Clade A were in Hardy-Weinberg equilibrium, while those in Clade C showed a significant deviation, indicating a departure from equilibrium. Clade A and C exhibited an *F*_IS_ value greater than 0, reflecting a complete lack of heterozygotes (homozygous excess). Additionally, the overall *F*_IS_ value for the population was also greater than 0, indicating an excess of homozygous individuals (Table 2).

**Table 2:** Distribution of *kdr* T917I mutation alleles in mitochondrial clades (A and C) of head louse populations.

Population	*n*	*kdr* genotypes			Resistance allele frequency	Hardy-Weinberg equilibrium		*F*_IS_^c^
		RR, *n* (%)	RS, *n* (%)	SS, *n* (%)		*χ*^2^	*P*-value	
Clade A	74	70 (94.59)	4 (5.41)	0 (0)	0.97	0.0571	*P* = 0.8111^a^	0.071
Clade C	33	31 (93.94)	0 (0)	2 (6.06)	0.94	33.000	*P* < 0.0001^b^	1.000
Both clades	107	101 (94.39)	4 (3.74)	2 (1.87)	0.96	24.713	*P* < 0.0001^b^	0.513

*Abbreviations*: RR, homozygous resistant; RS, heterozygous; SS, homozygous susceptible; *n*, number of head lice tested.

a The population is in Hardy-Weinberg equilibrium (*P* > 0.05; χ^2^ < 3.84).

b The population departs from Hardy-Weinberg equilibrium (*P* < 0.05; χ^2^ > 3.84).

c *F*_IS_ values > 0 indicate homozygous excess, while *F*_IS_ values < 0 indicate homozygous deficiency.

**Fig. 6:**
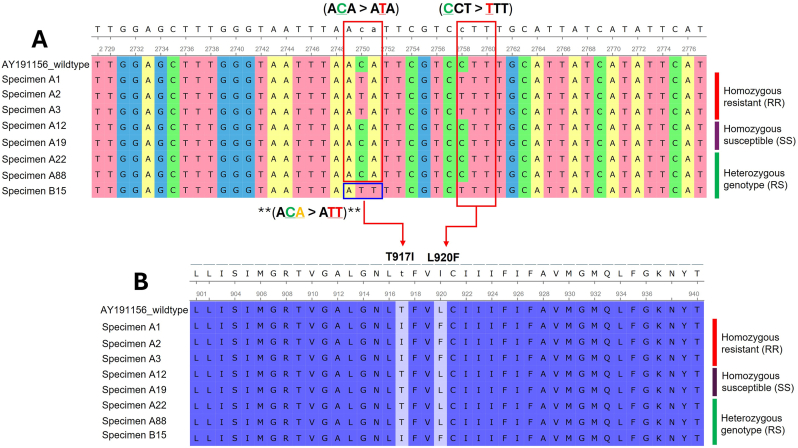
Alignment of nucleotide (**A**) and corresponding amino-acid sequences (**B**) of the α-subunit of the *VSSC* gene from head lice collected in this study, illustrating the RR (homozygous resistant), SS (homozygous susceptible), and RS (heterozygous) genotypes. The alignment highlights the positions of the established *kdr* T917I mutation (A**C**A > A**T**A) and the L920F mutation (**C**TT > **T**TT). Asterisks (∗∗) and the blue box indicate a novel T917I variant (A**CA** > A**TT**).

The RR genotypes were distributed in almost all haplotypes except H9 (Clade A). In contrast, the RS genotypes were identified in H1 (Clade A) and H9 (Clade A). Additionally, the SS genotypes were observed in two haplotypes, specifically H2 and H4, within Clade C (Table 3). In this study, representative samples of each RFLP genotype (RR, RS, and SS) were randomly selected for sequencing to confirm the observed patterns. The consensus nucleotide and amino-acid sequences were aligned and compared with the reference wild-type sequence of *Pediculus humanus capitis* (GenBank: AY191156), which includes the three known *kdr* mutations: M815I, T917I, and L920F. Sequence comparison revealed no substitution at codon T917 in samples with the homozygous susceptible (SS) genotype, consistent with the presence of the wild-type allele. In contrast, all homozygous resistant (RR) samples exhibited a non-synonymous point mutation at codon T917 (A**C**A > A**T**A), resulting in a threonine-to-isoleucine substitution (Thr > Ile), and consistently carried the L920F mutation (**C**TT > **T**TT; Leucine (Leu) > Phenylalanine (Phe)). Heterozygous (RS) sequences contained both the wild-type allele (without substitution at codon T917) and the mutant alleles harboring T917I and L920F mutations. Notably, one heterozygous sequence (code B15) displayed a novel T917I variant (A**CA** > A**TT**), differing from the previously reported A**C**A > A**T**A mutation; however, both mutations result in the same amino-acid substitution (Thr > Ile) (Fig. 6).

**Table 3:** Frequency (*n*) of haplotypes, *kdr* genotypes (T917I), and *Acinetobacter* spp. identified in the present study (*n* = 107).

Clade	Haplotype	*n*	*kdr* genotypes			*rpoB*-PCR
			RR	RS	SS	*Acinetobacter* spp.
A	H1	65	62^a^	3	0	*Acinetobacter towneri* (*n* = 1)
C	H2	16	15^a^	0	1	*Acinetobacter johnsonii* (*n* = 1)
C	H3	1	1	0	0	
C	H4	4	3	0	1	
A	H5	5	5^a^	0	0	*Acinetobacter* sp. (*n* = 1)
C	H6	7	7	0	0	
A	H7	1	1	0	0	
A	H8	1	1	0	0	
A	H9	1	0	1	0	
A	H10	1	1	0	0	
C	H11	1	1	0	0	
C	H12	1	1	0	0	
C	H13	1	1	0	0	
C	H14	1	1	0	0	
C	H15	1	1	0	0	

*Abbreviations*: RR, homozygous resistant; RS, heterozygous; SS, homozygous susceptible.

a Positive for *Acinetobacter* spp. detection.

### 3.4 Detection of bacterial pathogens in head lice DNA

In this study, PCR amplification targeting *Bartonella* spp. and *Acinetobacter* spp. was conducted on 107 head lice DNA samples. *Acinetobacter* DNA was detected in 3 samples (2.8%). BLAST analysis of the obtained nucleotide sequences revealed high similarity to known *Acinetobacter* species in the GenBank database: one matched *A. towneri* with 99.16% identity (GenBank: CP071771), another matched *A. johnsonii* with 99.58% identity (GenBank: CP059080), and the third sequence showed 100% identity with an unidentified *Acinetobacter* sp. (GenBank: CP028561). These *Acinetobacter*-positive lice were identified as H1 (Clade A), H2 (Clade C), and H5 (Clade A) (Table 3), and all carried the *kdr* T917I mutation. No *Bartonella* spp. DNA was detected in any of the head lice samples analyzed.

The raw data for the present study are provided in Supplementary file S2.

## 4 Discussion

This study comprehensively examines the genetic diversity of mitochondrial *cytb* sequences, knockdown resistance (*kdr*) mutations, and associated bacterial pathogens in head lice collected from orphanage children in Ang Thong Province, Thailand. Phylogenetic reconstruction and species delimitation using the ASAP and mPTP methods identified two distinct mitochondrial *cytb* clades, A and C, among the lice samples. Clade A was more prevalent, comprising 69.16% of the samples analyzed. This finding aligns with previous studies that reported Clade A as the most widespread lineage globally (Light et al., 2008; Boutellis et al., 2014; Ashfaq et al., 2015; Amanzougaghene et al., 2019b; Mirzajanzadeh et al., 2024). In contrast, Clade C has been documented primarily in parts of Africa (Ethiopia, Senegal, Gabon, and the Democratic Republic of the Congo), Asia (Nepal, Pakistan, Malaysia, and Thailand), and Europe (France) (Light et al., 2008; Boutellis et al., 2014; Ashfaq et al., 2015; Amanzougaghene et al., 2019b; Phadungsaksawasdi et al., 2021), suggesting that this lineage may represent a geographically restricted clade with potential regional specificity to Thailand. The identification of head lice clades A and C was supported by a high level of interspecific genetic divergence, ranging from 9.21 to 14.08%, which exceeds the commonly accepted threshold of 3% for species-level differentiation (Hebert et al., 2003). Additionally, nucleotide polymorphisms characterized by transition mutations further supported the distinction between the two clades (Phadungsaksawasdi et al., 2021).

The genetic diversity observed in head lice populations from this study was characterized by high haplotype diversity and relatively low nucleotide diversity, a pattern commonly associated with recent population divergence (Grant and Bowen, 1998). The haplotype network analysis, compared with previously reported *cytb* sequences from head lice in Thailand, revealed a star-like distribution pattern. This network displayed central ancestral haplotypes linked to multiple closely related haplotypes, with 1–4 base differences from Haplotype 1 (clade A), 1–2 base differences from Haplotype 14 (clade A), and 1–3 base differences from haplotype 34 (Clade C). These findings suggest a recent population expansion originating from ancestral haplotypes (Grant and Bowen, 1998; Phadungsaksawasdi et al., 2021). Haplotypes 1, 14, and 34 (in both clades, A and C) were widespread in the country, suggesting an ancestral history in Thailand. A study conducted by Phadungsaksawasdi et al. (2021) identified 50 novel haplotypes of head lice among primary school children across 15 provinces in Thailand, which suggests a potential historical link between Thai head lice populations and those from South Asia. Additionally, neutrality tests provided further insight into the demographic history of the populations. According to haplotype networks based on 381 *cytb* sequences among samples of Thailand, Tajima’s *D* value was not statistically significant, indicating no strong evidence of selection or recent demographic shifts. Conversely, Fu and Li’s *D* test yielded a significantly negative value, suggesting potential signals of recent population expansion or the action of purifying selection.

Analysis of genetic diversity and haplotype networks based on 107 *cytb* sequences of the present study identified 15 distinct haplotypes distributed across two mitochondrial clades. Neutrality tests revealed significantly positive values for both Tajima’s *D* and Fu and Li’s *D*, suggesting the presence of balancing selection or underlying population structure within the head lice populations. These patterns may reflect factors such as the admixture of divergent subpopulations or a historical reduction in population size. However, previous studies in Thailand have reported a general increase in head lice population size, although evidence of localized population bottlenecks has also been observed in certain regions (Yingklang et al., 2021).

Recent global surveillance indicates a significant increase in knockdown-resistance (*kdr*) mutations among head lice populations. Resistance-associated alleles such as T917I, L920F, and M815I now exceed a 50% frequency in many regions, which poses a threat to the effectiveness of pyrethroid-based treatments (Eslami et al., 2024; Abbasi et al., 2023). Although the *kdr* genotype does not invariably predict clinical pyrethroid resistance, Brownell et al. (2024) observed that head lice infestations lacking *kdr* mutations were associated with more favorable treatment outcomes compared with those carrying at least one resistant allele (RS or RR) (Bialek et al., 2011; Brownell et al., 2024). These observations support the concept that *kdr* mutations contribute substantially to the manifestation of pyrethroid resistance in head lice populations.

In this study, the *kdr* mutation was identified in 96% of the samples in both clades, which is a significant increase from the previous reports from Thailand. The prevalence of this resistance allele has increased significantly in central regions of the country, from 26 to 63% and ultimately to 96%, indicating its rapid and nearly complete fixation (Brownell et al., 2020, 2024). The observed distribution of *kdr* resistance alleles in Clade C deviated from the Hardy-Weinberg equilibrium, suggesting that evolutionary forces are occurring in these head louse populations collected in the orphanage. A positive inbreeding coefficient (*F*_IS_ > 0) indicated an abundance of homozygous resistant genotypes. This trend indicates significant selective pressure from recurrent pyrethroid exposure, promoting the retention of resistance alleles in head louse populations. These findings suggest an adaptive shift induced by insecticidal pressure and emphasize the necessity of resistance monitoring and alternative control measures (Brownell et al., 2020; Larkin et al., 2020).

All homozygous resistant (RR) head lice exhibited the T917I (A**C**A > A**T**A) and L920F (**C**TT > **T**TT) mutations, thereby affirming their association with pyrethroid resistance, while the heterozygous (RS) genotype displayed partial resistance. Notably, one heterozygous sequence possessed a novel T917 variant (A**CA** > A**TT**) that resulted in the same Thr > Ile substitution, indicating that different nucleotide pathways can yield equivalent functional results. This underscores the evolutionary adaptability of *kdr* alleles and emphasizes the necessity to monitor both established and alternative mutations to understand resistance dynamics comprehensively.

Head lice are known to carry DNA from medically important bacteria, including *Acinetobacter* spp., *Staphylococcus aureus*, *Serratia marcescens*, *Bartonella quintana*, *Coxiella burnetii*, and *Rickettsia aeschlimannii* (Sunantaraporn et al., 2015; Amanzougaghene et al., 2017; Mokhtar et al., 2020; Dzul-Rosado et al., 2022). Studies from Malaysian welfare homes and disadvantaged communities in Mexico reported bacterial DNA of *Acinetobacter* spp. in 24–43% of head lice (Mokhtar et al., 2020; Dzul-Rosado et al., 2022). In this study, *Acinetobacter* spp. DNA was found at a low prevalence (2.8%) in head lice from children living in an orphanage. This finding implies that local environmental conditions may influence bacterial carriage, which may vary among various populations and geographical areas. Specifically, DNA of *Acinetobacter towneri*, *Acinetobacter* sp., and *Acinetobacter johnsonii* was identified in three head lice samples. All head lice samples that tested positive for pathogens exhibited a homozygous resistant (RR) genotype across both clades A and C (Haplotypes H1, H2, and H5).

Molecular investigations worldwide have consistently detected *Acinetobacter* DNA in human head lice, with *Acinetobacter baumannii* being the most common multidrug-resistant opportunistic pathogen found in regions such as Thailand, Algeria, the Democratic Republic of the Congo, Mexico, and Latin America (Bouvresse et al., 2011; Kempf et al., 2012; Sunantaraporn et al., 2015; Mana et al., 2017; Boumbanda Koyo et al., 2019; Mokhtar et al., 2020; Dzul-Rosado et al., 2022; Larkin et al., 2023). Additional pathogenic species, including *A. pittii*, *A. nosocomialis*, *A. schindleri*, and *A. johnsonii*, have also been documented, while environmental species, including *A. towneri*, *A. variabilis*, *A. soli*, *A. radioresistens*, and *A. guillouiae*, appear more sporadically (Sunantaraporn et al., 2015; Mana et al., 2017; Amanzougaghene et al., 2019b; Boumbanda Koyo et al., 2019; Mokhtar et al., 2020). The identification of *A. johnsonii* in head lice is significant, given that this species, which was historically thought of as a low-virulence environmental organism, has been implicated in serious infections, including a reported case of meningitis in a 15-year-old patient (Gutiérrez-Gaitán et al., 2022). These findings indicate that the pathogenic potential of *Acinetobacter* bacteria is not limited to *A. baumannii*. The ongoing detection of *Acinetobacter* DNA in head lice suggests that these organisms could serve as reservoirs or mechanical carriers for these opportunistic pathogens. Species of *Acinetobacter* are known to colonize human skin, persist on fomites, and thrive in diverse environmental niches (Veracx and Raoult, 2012). Their acquisition by head lice may therefore occur through multiple routes, including blood-feeding on colonized hosts or incidental contact with contaminated surroundings. This ecological overlap may explain why head lice consistently harbor a mixture of both pathogenic and environmental strains. While the detection of DNA does not establish vector competence, the consistent detection of *Acinetobacter* DNA in head lice populations prompts critical inquiries into their epidemiological implications.

From a public health perspective, the detection of bacterial DNA in head lice suggests that these ectoparasites may signify more than simple nuisances. They may be associated with the spread of clinically significant opportunistic bacteria. It is imperative to expand our focus beyond *A. baumannii* to include other species, such as *A. johnsonii*, which may pose epidemiological risks. Improving surveillance programmes focused on microbial detection and insecticide resistance is crucial for developing effective control strategies and guiding public health interventions, especially for vulnerable populations prone to head lice infestations.

The primary limitation of this study is the restricted sampling scope, as head lice specimens were collected from a single orphanage site. The complete distribution or genetic diversity of head lice populations throughout Thailand may not be accurately represented by these data. Additionally, the pathogen screening was limited to *Acinetobacter* spp. and *Bartonella* spp., which are bacteria that are generally associated with head lice. This may have resulted in the exclusion of other, less common, or previously unidentified pathogens. Additionally, the low prevalence of bacterial pathogens and the predominance of homozygous resistant genotypes rendered it impossible to assess the potential correlations between head lice genetic clades, pathogen detection, and *kdr* genotypes, as the presence of heterozygous or wild-type genotypes was negligible. The methodology and conceptual framework of this research focused on detecting and assessing the prevalence of *kdr* mutations in head lice among a designated population of orphanage children. However, the findings are subject to certain limitations. The underlying cause of the high frequency of *kdr* mutations in this population remains uncertain. This phenomenon may result from selective pressure associated with prolonged pyrethroid exposure, gene flow through the migration of *kdr*-carrying lice within the orphanage, or the introduction of resistant lice from external sources. As a result, future research should implement a more comprehensive sampling framework that includes orphanages from a variety of geographical regions in Thailand and expand molecular screening to encompass a broader range of medically significant bacterial taxa. This approach would offer a more thorough comprehension of the dynamics of resistance and pathogen diversity in head lice populations.

## 5 Conclusions

This study provides new insights into the genetic structure, insecticide resistance, and potential bacterial associations of head lice collected from children aged 7–12 years at an orphanage in Ang Thong Province, central region of Thailand. Mitochondrial *cytb* analysis revealed two distinct clades, A and C, indicating the coexistence of genetically divergent lineages within the population. The widespread detection of the *kdr* mutation in 96% of samples across both clades suggests a high level of resistance to commonly used pyrethroid-based pediculicides, raising concerns about the continued efficacy of conventional treatment methods. Moreover, the identification of *Acinetobacter* spp. in head lice samples highlights the potential role of head lice as vectors or reservoirs of bacterial pathogens. Although the clinical significance of these bacteria in head lice remains to be fully elucidated, their presence warrants attention, particularly in vulnerable populations with limited access to healthcare and hygiene resources. These findings emphasize the need for continuous molecular surveillance of both head lice genetic diversity and insecticide resistance markers to inform more effective control strategies. Furthermore, the detection of *Acinetobacter* species suggests a need for additional research into the vectorial capacity of head lice and their role in the transmission of bacterial pathogens. Public health interventions should not only focus on improving head lice treatment protocols but also incorporate education and monitoring programmes, particularly in high-risk group settings such as orphanages and primary schools.

## CRediT authorship contribution statement

**Urooj Gul:** Methodology, Investigation, Formal analysis, Writing – original draft. **Sakone Sunantaraporn:** Methodology, Formal analysis, Writing – original draft. **Padet Siriyasatien:** Data curation, Validation, Resources, Supervision, Writing – review & editing. **Narisa Brownell:** Conceptualization, Data curation, Methodology, Formal analysis, Project administration, Validation, Writing – review & editing.

## Ethical approval

This project was approved and reviewed by the Institutional Review Board of the Faculty of Medicine, Chulalongkorn University, Bangkok, Thailand (COA. 0758/2025). All participants received a thorough explanation of the study, and written informed consent was obtained from their legal guardian.

## Statement on the use of AI-assisted technologies

During the preparation of this work, the authors used Grammarly (https://www.grammarly.com/) in order to correct grammatical errors and improve readability. After using this tool, the authors reviewed and edited the content as needed and take full responsibility for the content of the published article.

## Funding

This research project was supported by the Ratchadapiseksompotch Fund, Faculty of Medicine, Chulalongkorn University (Grant number GA 69/040).

## Declaration of competing interests

The authors declare that they have no known competing financial interests or personal relationships that could have appeared to influence the work reported in this paper.

## Data Availability

All data generated or analyzed during this study are included in this published article and its supplementary files. The newly generated sequences were submitted to the GenBank database under the accession numbers PX505907-PX505921 (*cytb*), PX505922-PX505929 (*VSSC*), PX505930 (*Acinetobacter johnsonii*), PX505931 (*Acinetobacter* sp.), and PX505932 (*Acinetobacter towneri*).
