# Supplementary material for: High prevalence of knockdown resistance mutations, genetic clade diversity, and detection of Acinetobacter species in head lice (Pediculus humanus capitis) infesting children in a Thai orphanage: A comprehensive survey
Source: Curr Res Parasitol Vector Borne Dis. 2025 Nov 17;8:100336. doi: 10.1016/j.crpvbd.2025.100336 (PMC12681526; doi:10.1016/j.crpvbd.2025.100336)
Supplement: Multimedia component 1 [file mmc1.pdf]

Supplementary file 1

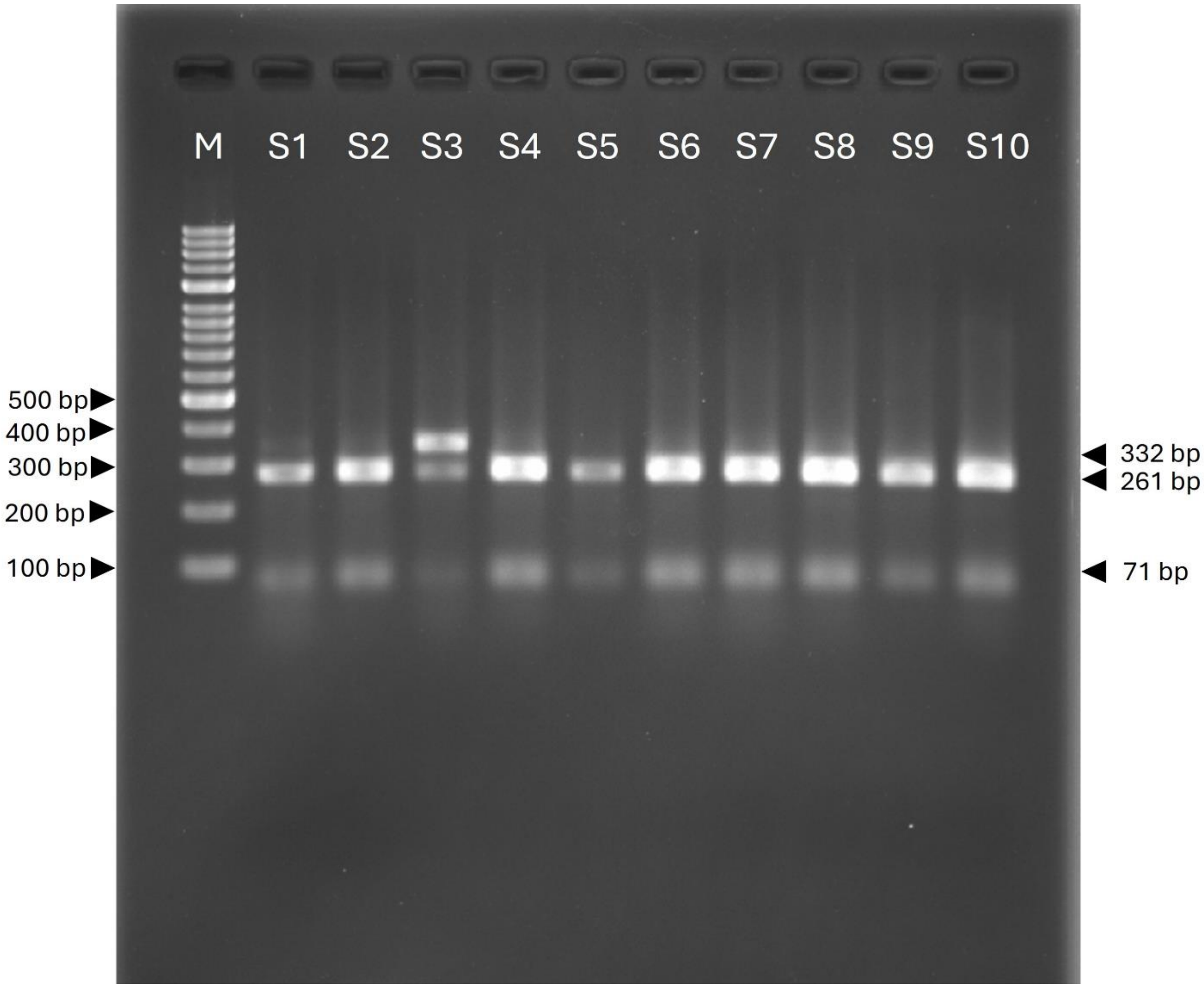

Supplementary Figure S1. 2% agarose gel electrophoresis showing RFLP patterns of the *kdr* T917I genotypes. Lane S3 represents the heterozygous (RS) genotype with three bands, while Lanes S1, S2, and S4–S10 show the homozygous resistant (RR) genotype with two bands. Lane M: 100 bp DNA marker.
