# Supplementary material for: High prevalence of knockdown resistance mutations, genetic clade diversity, and detection of Acinetobacter species in head lice (Pediculus humanus capitis) infesting children in a Thai orphanage: A comprehensive survey
Source: Curr Res Parasitol Vector Borne Dis. 2025 Nov 17;8:100336. doi: 10.1016/j.crpvbd.2025.100336 (PMC12681526; doi:10.1016/j.crpvbd.2025.100336)
Supplement: Multimedia component 2 [file mmc2.pdf]

Supplementary file 2

Summary of the number of head lice, mitochondrial clade classification, and *kdr* genotypes.

No.	Code	Stage	Clade	Haplotype	*kdr* genotype
1	A1	Female	A	H1	RR
2	A2	Female	A	H1	RR
3	A3	Female	A	H1	RR
4	A4	Male	A	H1	RR
5	A5	Nymph	C	H2	RR
6	A6	Male	A	H1	RR
7	A7	Male	C	H3	RR
8	A8	Male	A	H1	RR
9	A9	Male	A	H1	RR
10	A10	Male	A	H1	RR
11	A11	Male	A	H1	RR
12	A12	Male	C	H4	SS
13	A13	Male	A	H5	RR
14	A14	Nymph	C	H2	RR
15	A15	Nymph	A	H1	RR
16	A16	Female	C	H2	RR
17	A17	Male	A	H1	RR
18	A18	Female	A	H1	RR
19	A19	Nymph	C	H2	SS
20	A20	Female	A	H5	RR
21	A21	Female	C	H6	RR
22	A22	Female	A	H1	RS
23	A63	Female	A	H1	RR
24	A64	Female	C	H2	RR
25	A65	Female	A	H5	RR
26	A66	Female	A	H1	RR
27	A67	Female	A	H1	RR
28	A68	Male	A	H5	RR
29	A69	Male	A	H1	RR
30	A70	Female	A	H7	RR
31	A71	Male	C	H2	RR
32	A72	Female	A	H1	RR
33	A73	Female	A	H1	RR
34	A74	Female	C	H2	RR
35	A75	Female	A	H1	RR
36	A76	Male	A	H1	RR
37	A78	Male	A	H1	RR
38	A79	Male	A	H1	RR
39	A80	Female	A	H1	RR
40	A81	Nymph	A	H1	RR
41	A82	Female	A	H1	RR
42	A83	Male	A	H1	RR
43	A84	Male	C	H2	RR
44	A85	Female	A	H1	RR
45	A86	Female	C	H2	RR
46	A87	Male	A	H8	RR
47	A88	Female	A	H9	RS
48	A89	Nymph	A	H1	RR
49	A90	Male	C	H2	RR
50	A91	Female	A	H10	RR

No.	Code	Stage	Clade	Haplotype	*kdr* genotype
51	A92	Female	C	H2	RR
52	A93	Female	A	H1	RR
53	A94	Nymph	A	H1	RR
54	A95	Male	A	H1	RR
55	A96	Nymph	A	H1	RR
56	A97	Male	C	H2	RR
57	A98	Male	A	H1	RR
58	A99	Female	A	H1	RR
59	A100	Female	A	H1	RR
60	B1	Nymph	C	H2	RR
61	B2	Female	A	H1	RR
62	B3	Nymph	A	H1	RR
63	B4	Male	A	H1	RR
64	B5	Female	C	H2	RR
65	B6	Male	C	H2	RR
66	B7	Female	A	H1	RR
67	B8	Female	A	H1	RR
68	B9	Female	A	H1	RR
69	B10	Female	C	H2	RR
70	B11	Female	A	H1	RR
71	B13	Female	A	H1	RR
72	B14	Female	A	H1	RR
73	B15	Male	A	H1	RS
74	B17	Male	C	H6	RR
75	B18	Female	A	H1	RR
76	B19	Nymph	A	H1	RR
77	B20	Female	C	H14	RR
78	B21	Male	C	H6	RR
79	B22	Nymph	A	H1	RR
80	B23	Male	C	H15	RR
81	B24	Male	A	H5	RR
82	B25	Male	A	H1	RR
83	B26	Male	A	H1	RR
84	B27	Nymph	A	H1	RS
85	B28	Male	A	H1	RR
86	B29	Nymph	C	H11	RR
87	B30	Female	A	H1	RR
88	B32	Male	C	H6	RR
89	B33	Male	C	H12	RR
90	B34	Male	C	H6	RR
91	B35	Male	C	H4	RR
92	B36	Nymph	A	H1	RR
93	B37	Female	A	H1	RR
94	B38	Female	A	H1	RR
95	B39	Male	A	H1	RR
96	B41	Male	A	H1	RR
97	B42	Female	A	H1	RR
98	B43	Male	C	H4	RR
99	B44	Female	C	H4	RR
100	B45	Nymph	C	H13	RR
101	B46	Male	A	H1	RR
102	B47	Male	A	H1	RR
103	B48	Male	A	H1	RR
104	B49	Male	A	H1	RR

No.	Code	Stage	Clade	Haplotype	*kdr* genotype
105	B50	Female	A	H1	RR
106	B51	Male	C	H6	RR
107	B52	Male	C	H6	RR

Abbreviations: RR: homozygous resistant genotype; SS, homozygous susceptible genotype; RS, heterozygous genotype.

Table 2: Haplotype distribution of *cytb* sequences and their GenBank accession numbers

Accession number	Haplotype	No. of sequences
PX505907	H1	65
PX505908	H2	16
PX505909	H3	1
PX505910	H4	4
PX505911	H5	5
PX505912	H6	7
PX505913	H7	1
PX505914	H8	1
PX505915	H9	1
PX505916	H10	1
PX505917	H11	1
PX505918	H12	1
PX505919	H13	1
PX505920	H14	1
PX505921	H15	1

Table 3: Accession number information for *VSSC* gene sequences and identified *Acinetobacter* species.

Accession number	Genes	Identification	*kdr* genotype
PX505922	*VSSC*	Specimen A1	RR
PX505923	*VSSC*	Specimen A2	RR
PX505924	*VSSC*	Specimen A3	RR
PX505925	*VSSC*	Specimen A12	SS
PX505926	*VSSC*	Specimen A19	SS
PX505927	*VSSC*	Specimen A22	RS
PX505928	*VSSC*	Specimen A88	RS
PX505929	*VSSC*	Specimen B15	RS
PX505930	*rpoB*	*Acinetobacter johnsonii*	-
PX505931	*rpoB*	*Acinetobacter* sp.	-
PX505932	*rpoB*	*Acinetobacter towneri*	-

RR: homozygous resistant, SS: homozygous susceptible, and RS: heterozygous genotypes.
